# Four-dimensional flow cardiovascular magnetic resonance for the assessment of mitral stenosis

**DOI:** 10.1093/ehjcr/ytab465

**Published:** 2021-11-19

**Authors:** James Wardley, Andrew Swift, Alisdair Ryding, Pankaj Garg

**Affiliations:** 1 Cardiology, Norfolk and Norwich University Hospital NHS Trust, Colney Lane, NR4 7UY, Norwich, UK; 2 Cardiovascular and Metabolic Health, Norwich Medical School, Bob Champion Research & Education Building, University of East Anglia, NR4 7UQ, Norwich, UK; 3 Department of Infection, Immunity & Cardiovascular Disease, University of Sheffield, S10 2RX, Sheffield, UK

**Figure 1:**
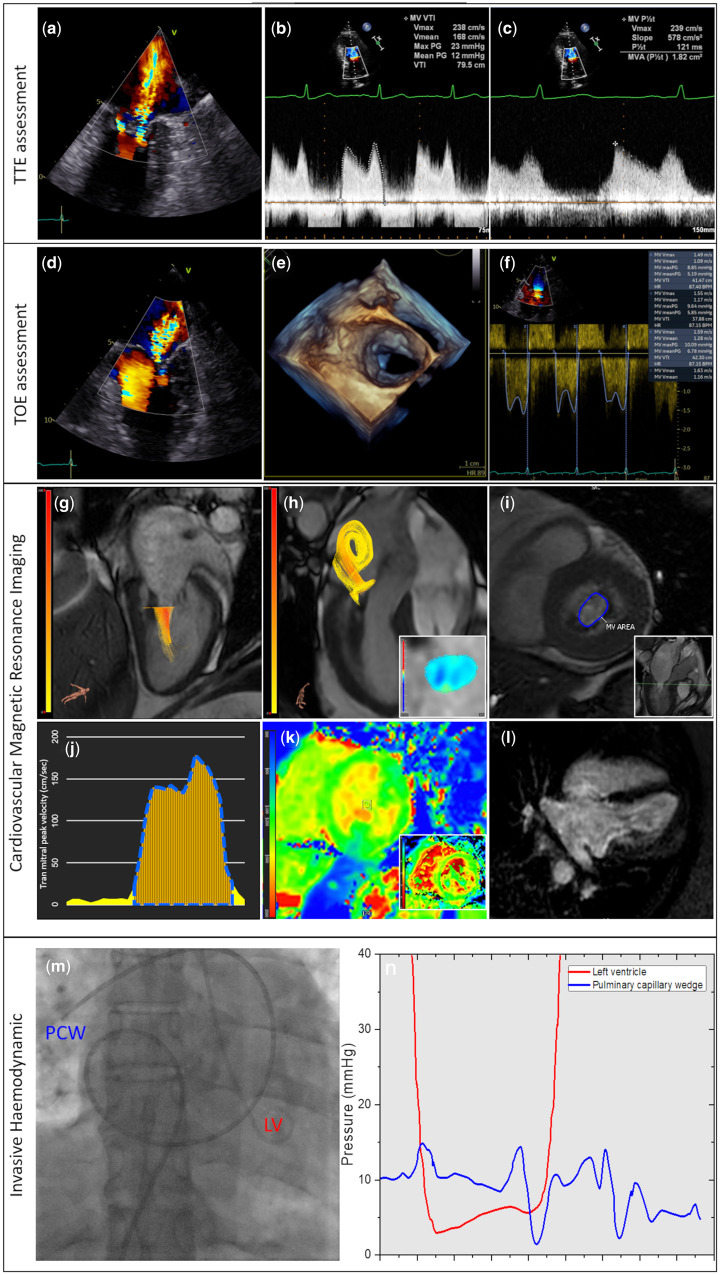
Multi-modality assessment of mixed mitral valve disease. Transthoracic echocardiography: Turbulent mitral regurgitation demonstrated in the apical two chamber with dilated left atrium (*A*). Velocity time integral-derived mean pressure gradient through the mitral valve (*B*) was 12 mmHg and the pressure half-time (*C*) estimated the mitral valve area to be 1.82 cm^2^. On transoesophageal echocardiography, severe mitral regurgitation is seen in two-chamber (*D*) and on three-dimensional reconstruction of the mitral valve (*E*) the degree of mitral stenosis does not appear to be severe. On velocity time integral (*F*), the mean pressure gradient was 6 mmHg. On four-dimensional flow cardiovascular magnetic resonance, the peak mitral inflow velocity was traced throughout the diastole using plane with maximum velocity (*G*) and during systole (*H*), a reformatted plane was generated perpendicular to the mitral regurgitation jet in the left atrium (*H*). On short-axis cine, mitral valve area was 2.1 cm^2^ at the tips of mitral valve (*I*). The mean pressure gradient for mitral inflow was 6 mmHg (*J*). T1-mapping (*K*) and extracellular mapping demonstrated normal myocardium. There was no evidence of left ventricular scar on late gadolinium enhancement (*L*). Invasive haemodynamic assessment was done using a multipurpose catheter placed for the capillary wedge pressure and a pigtail catheter within the left ventricle to allow recording of simultaneous pressures (*M*). The simultaneous pressure tracings of left ventricle and pulmonary capillary wedge are demonstrated (*N*).

A 52-year-old lady presented with breathlessness with no physical stigmata of heart failure. Transthoracic echocardiography demonstrated a rheumatic-looking mitral valve (MV) with a mean pressure gradient (PG) of 12 mmHg, a dilated left atrium (32 cm^2^), and moderate mitral regurgitation (MR) (*Figure 1A*–*C*) (*Video 1*). On transoesophageal echocardiography (TOE), the mean PG was 7 mmHg and the MR was severe with a PISA of 0.1–1.02 cm (*Figure 1D*–*F*). At this stage, the regional heart team suggested further assessment by invasive haemodynamic and cardiovascular magnetic resonance (CMR). For CMR, in addition to the standard assessment, four-dimensional (4D) flow was done (*Figure 1G*–*L*) (*Video 1–2*). CMR cines demonstrated similar restrictive MV leaflets. On short-axis cines, the MV opening area at the tips of the leaflets was 2.1 cm^2^ and on 4D flow assessment the mean MV PG was 6 mmHg with only 13 mL of MR. Coronary angiography did not demonstrate any coronary disease and the invasive haemodynamic assessment was in keeping with the TOE with 6 mmHg mean PG (*Figure 1M* and *N*); the MV area was 2.1 cm^2^ by the Gorlin method. To summarize, TOE, invasive assessment, and CMR confirmed she had at most mild to moderate mitral stenosis (MS). CMR elucidated the MR severity to be mild. This case highlights several points: echocardiography can overestimate the regurgitation jet in restrictive valvular opening, mainly due to exaggeration of turbulent backward-flow through a restrictive MV; MR quantification by CMR can help decision-making in cases where echocardiographic methods are inconsistent; and as this case highlights for the first-time, 4D flow can quantify mean PG across the MV for MS assessment.

**Consent:** The authors confirm that written consent for submission and publication of this case report including images and associated text has been obtained from the patient in line with COPE guidance.

**Conflict of interest:** P.G. is clinical advisor to Pie Medical Imaging and Medis Medical Imaging.

**Funding:** This work was partly funded by Wellcome Trust grants (220703/Z/20/Z and 215799/Z/19/Z). For the purpose of open access, the author has applied a CC BY public copyright licence to any Author Accepted Manuscript version arising from this submission.

